# Treatment With Nilvadipine Mitigates Inflammatory Pathology and Improves Spatial Memory in Aged hTau Mice After Repetitive Mild TBI

**DOI:** 10.3389/fnagi.2018.00292

**Published:** 2018-10-11

**Authors:** Alexander Morin, Benoit Mouzon, Scott Ferguson, Daniel Paris, Nicole Saltiel, Carlyn Lungmus, Mike Mullan, Fiona Crawford

**Affiliations:** ^1^The Roskamp Institute, Sarasota, FL, United States; ^2^The Open University, Milton-Keynes, United Kingdom; ^3^James A Haley Veterans Administration, Tampa, FL, United States

**Keywords:** treatment, concussion, TBI, tau, brain injury, animal model, neuroinflammation, neurodegeneration

## Abstract

Mild traumatic brain injury (mTBI) is the most common form of brain trauma worldwide. The effects of mTBI are not well-studied within the elderly population, yet older adults constitute a significant portion of all mTBI patients. Few preclinical studies have focused on the effects of mTBI, or mTBI treatments, in the aged brain, and none have explored repetitive mTBI (r-mTBI). In this study, we have administered our well-characterized 5-injury model (5 r-mTBI) to hTau mice aged 24 months to explore the neurobehavioral and neuropathological outcomes, and the effects of treatment with the dihydropyridine, Nilvadipine. Our previous studies have shown that Nilvadipine inhibits spleen tyrosine kinase (Syk), is effective at reducing inflammation, tau hyperphosphorylation, and amyloid production, and it has recently been investigated in a European Phase III clinical trial for Alzheimer’s disease (AD). In our 24-month-old r-mTBI mice, we observed increased neuroinflammation and a trend toward impaired cognitive performance compared to sham controls. Treatment with Nilvadipine mitigated the TBI-induced inflammatory response in aged r-mTBI animals and significantly improved spatial memory. To our knowledge, this is the only preclinical study focusing on the treatment of r-mTBI in aged, and these results suggest a therapeutic potential of Nilvadipine for consequences of mTBI.

## Introduction

Mild traumatic brain injury (mTBI) is recognized as a major cause of disability in the USA and worldwide. Annually, 42 million people seek medical care due to mTBI (Gardner and Yaffe, 2015). TBI patients aged 65 and older have double the rate of hospitalizations compared to younger patients (<65 years: 60.6 per 1,00,000; >65 years: 155.9 per 1,00,000; Coronado et al., 2005), with ground falls and motor vehicle accidents (MVAs) as primary causes of brain trauma (Thompson et al., 2006; Mak et al., 2012). Age alone is known to be associated with cognitive decline and dementia, but also significantly worsens mTBI-related outcomes (Bartrés-Faz et al., 2001; LeBlanc et al., 2006; Thompson et al., 2006; Onyszchuk et al., 2008; Mak et al., 2012; Lee et al., 2013). Growing evidence links mTBI, and particularly repetitive mTBI (r-mTBI), with neurodegenerative diseases such as Alzheimer’s disease (AD; Lee et al., 2013; Tateno et al., 2015), Parkinson’s disease (PD; Jafari et al., 2013), amyotrophic lateral sclerosis (ALS; McKee et al., 2010), and chronic traumatic encephalopathy (CTE; McKee et al., 2013; Gardner and Yaffe, 2015). Persons aged 65 and older have a higher probability of developing these disabilities following mTBI due to pre-existing medical conditions, worsened functional recovery, and excessive pathological response (Mak et al., 2012). Additionally, the aged population is highly susceptible to repetitive brain injuries due to age-related deficits in motor functions, particularly balance (Langlois et al., 2003; Lajoie and Gallagher, 2004; Flanagan et al., 2006). A single mTBI increases the probability of future falls, leading to repetitive concussions; and the cumulative injury effect in the elderly is thought to lead to more severe cognitive and pathological outcomes from similar injuries than in the young (Rothweiler et al., 1998; Teo et al., 2018). To date, the majority of preclinical studies that have examined mTBI and r-mTBI have focused on young adult rodents, creating a void in the literature with respect to age and the role of co-morbid conditions at the time of injury (Shitaka et al., 2012; Petraglia et al., 2014a,b; Mierzwa et al., 2015; Zhang et al., 2015). Understanding the mechanisms following mTBI and r-mTBI in the elderly is an important undertaking.

To our knowledge, few pre-clinical studies have looked at the effect of mTBI on cognitive performance in aged mice, and they are limited to moderate and severe models of TBI only. One such study demonstrated that aged mice (21–24 months old) exposed to a controlled cortical impact (CCI) show greater impairments in cognitive and sensorimotor functions compared to young adult mice (5–6 months old; Onyszchuk et al., 2008). Moreover, the same study showed that only the older animals exhibit prolonged neurodegeneration and blood-brain barrier (BBB) disruption 2 months after injury. Microglial response and NADPH oxidase activity have also been shown to be elevated after CCI in aged animals and associated with inflammation and decreased antioxidant capacity, respectively (Kumar et al., 2013). Although these studies show a common trend in higher vulnerability of the aged brains to TBI, CCI is a severe injury, and thus these studies are not representative of concussion-related hospitalizations in an older human population, which are mostly due to mild or moderate injuries resulting from falls and MVAs (Thompson et al., 2006; Mak et al., 2012). The high incidence of geriatric mTBI and r-mTBI (Papa et al., 2012) necessitates greater attention to understanding TBI sequelae in the elderly and possible treatment opportunities.

The human neuropathology of r-mTBI has many facets which are presumed to share common mechanisms with neurodegenerative diseases. For example, r-mTBI and AD share similar pathological features such as the presence of neurofibrillary tangles (NFTs), a marked neuroinflammatory response, and to a lesser extent, the presence of amyloid-β (Aβ) pathology (Tateno et al., 2015). There are currently no FDA-approved drugs for the treatment of TBI, however, the shared pathology may indicate that AD treatments could be effective at treating the consequences of mTBI by reducing Aβ, tau hyperphosphorylation, and neuroinflammation. The anti-hypertensive drug, Nilvadipine, has been shown to enhance Aβ clearance, decrease tau phosphorylation, and reduce inflammation in a mouse model of AD (Paris et al., 2014). Paris et al. (2014) proposed that Nilvadipine suppresses all three of the aforementioned processes by inhibiting phosphorylated spleen tyrosine kinase (PSyk). Activated Syk phosphorylates tau directly or via GSK3β and activates NF-kB, leading to neuroinflammation and excessive BACE1 production, which is responsible for Aβ formation (Buggia-Prevot et al., 2008; Harris et al., 2012; Ly et al., 2013; Llorens-MarÃ­tin et al., 2014; Paris et al., 2014). Thus, Nilvadipine has the potential to be a multimodal AD treatment through the novel target of Syk; and a Phase III clinical trial of Nilvadipine in AD has recently been successfully completed in Europe (ClinicalTrials.gov: NCT02017340; Lawlor et al., 2014). Although our r-mTBI model does not demonstrate TBI-induced amyloid pathology in either wild type (WT) or hTau mice (Mouzon et al., 2014; Mouzon B. C. et al., 2018; Mouzon B. et al., 2018), neuroinflammation is a consistent feature of this model, and TBI-dependent effects on Syk phosphorylation and tau have been reported within 15 days of injury in both young and aged mice (Ferguson et al., 2017). Here, we describe a study in which aged mice received r-mTBI and were treated with Nilvadipine for 21 days thereafter to examine its effect on cognition, neuroinflammation, and tau pathology. We used transgenic hTau mice that express all six isoforms of human tau to better mimic r-mTBI outcomes associated with tau pathology and cognitive deficits (Andorfer et al., 2003). A battery of behavior tests and pathological analyses were performed assess r-mTBI outcomes in these animals and to evaluate the efficacy of treatment.

## Materials and Methods

### Animals

Male and female hTau mice (Jackson Laboratories, Bar Harbor, ME, USA) were aged at the Roskamp Institute until 82–106 weeks old (weight 19–32 g). The animals were housed under standard laboratory conditions (12-h light/dark cycle, 23 ± 1°C, 50 ± 5% humidity) with free access to food and water. All procedures were perform under the Roskamp Institutional Animal Care and Use Committee (IACUC) approval and AAALAC guidelines and in accordance with the National Institutes of Health Guide for the Care and Use of Laboratory Animals.

### Experimental Groups and Study Design

**Figure 1:**
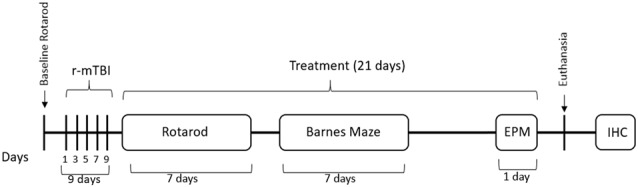
Outline of the study design. Mice received five injuries over a 9 day period with a 48-h interval between the injuries. A battery of behavior tests followed the last injury and included Rotarod (7 days), barnes maze (BM; 7 days) and elevated plus maze (EPM) on the last day before euthanasia. Mice received treatment of Nilvadipine injections for a 21-day period, starting immediately after the last injury. Mice were euthanized on day 22 after the last injury.

A total of 39 mice were randomly assigned to one of four groups: sham/vehicle (*n* = 9), sham/Nilvadipine (*n* = 9), r-mTBI/vehicle (*n* = 10), r-mTBI/Nilvadipine (*n* = 11). Each group included both male and female mice. All animals underwent anesthesia for the same duration and frequency. Sham animals were allowed to recover in their home cages after each anesthesia, while r-mTBI mice received five injuries over 9 days with a 48-h inter-concussion interval, a well-established injury paradigm (Mouzon et al., 2012, 2014; Mouzon B. et al., 2018; Tzekov et al., 2014, 2016; Ojo et al., 2015). Either Nilvadipine or vehicle (phosphate-buffered saline (PBS): Polyethylene glycol (PEG) 1:1) were injected intraperitoneally (i.p.) daily for 21 days, with the first injection administered immediately following the last anesthesia (in sham) or the last injury (in r-mTBI). Behavior tests started on the second day of injections and included Rotarod, Barnes Maze (BM), and elevated plus maze (EPM), as shown in Figure 1. Mice were euthanized 24 h after the last injection (22 days after the last injury/anesthesia). Brains were further analyzed by immunohistochemical and biochemical methods for inflammatory markers, tau, and phosphorylated Syk. Researchers were blind to animal group assignments during both neurobehavioral experiments and immunohistochemistry.

### Injury Protocol

mTBI/sham injury was performed as previously described (Mouzon et al., 2012, 2014; Mouzon B. et al., 2018; Tzekov et al., 2014, 2016; Ojo et al., 2015). All animals were anesthetized with 1.5 L/min of oxygen and 3% isoflurane prior to r-mTBI or sham injury. The heads were shaved, and mice were placed on a heating pad to maintain body temperature at 37°C to prevent hypothermia. The head of each animal was fixed in a stereotaxic frame, and the blunt impactor tip (3 mm diameter) was positioned midway to the sagittal suture. The injury was triggered at 5 m/s velocity and 1.0 mm depth, with a dwell time of 200 ms, using an Impact One™ stereotaxic impactor (Leica Microsystems, Richmond, IL, USA). All mice experienced short-term apnea (<20 s) and showed no skull fractures. All animals were allowed to recover from anesthesia on a heating pad and then returned to their cages with water and soft food access. Sham animals received anesthesia alone for the same duration of time as the r-mTBI mice to control for the effects of repeated anesthesia. Mice were monitored daily for any abnormalities in behavior.

### Treatment

All mice received either Nilvadipine or vehicle via i.p. injections for 21 days, starting immediately after the last injury (the first injection was administered while animals were still under anesthesia). The site for i.p. injection was sterilized with alcohol and was changed every day to avoid discomfort due to repeated injections at the same site. The treated groups received 2 mg/kg Nilvadipine (a physiologically relevant dose conferring antihypertensive activity) dissolved in a 1:1 solution of PBS and PEG vehicle solution (Paris et al., 2014). The injection volume (100 μl) was calculated based on the average animal’s weight (0.028 kg). Untreated animals underwent the same procedure, but received vehicle solution only (PBS:PEG/1:1). The Nilvadipine and vehicle solutions were freshly prepared every day before the injections.

### Motor Function Assessment

Motor function was assessed using the Rotarod apparatus, and the latency to fall from an accelerating rotating rod was measured. Baseline performance was recorded 1 day prior to the first injury/sham. Rotarod assessment started on the next day after the last injury/sham procedure and was perform on days 1, 3, 5 and 7 post-last r-mTBI. An acclimation period involved three trials with a duration of 3 min each and a 3 min rest interval in the animal’s home cage between the trials (velocity = 5 rpm, no acceleration). The mice were placed back on the bar during the acclimation period if they fell. All experimental trials, including the baseline trials, lasted for 5 min and were conducted with acceleration from 5 rpm to 50 rpm over the 5 min period. Each animal underwent three trials per day, with a 3 min rest interval between each trial. The fall time of each mouse was recorded in seconds. To ensure that fall time would correlate with motor coordination rather than purely grip strength, if a mouse clung to the bar for more than five consecutive rotations on the accelerating rod without walking or making forward progress against the rotation of the bar, the time of the 5th rotation was considered to be the fall time and was recorded as such.

### Cognitive Function Assessment

BM was initiated on day 8 post-last mTBI/sham and lasted for seven consecutive days to assess cognitive function. For 6 days, animals were trained to find the target hole which had a black escape box underneath. The walls in the room were equipped with visual cues and the brightness of the room was consistent throughout testing (7 days). The BM table is 1.2 meters in diameter and has 18 equally spaced holes around the perimeter. Every mouse had four acquisition trials per day, with a duration of 1.5 min each. The starting position for each trial during acquisition rotated, beginning at one of four cardinal directions of the maze and rotating, first clockwise (until reaching the initial position again) and then counterclockwise. If an animal did not find the target hole or did not go inside the box within the time limit, the mouse was guided to the target hole by hand. Regardless of their success, mice then spent 30 s in the box before returning to their cage. On the last day, 24 h following the final acquisition trial, a probe trial was conducted during which the animals were placed in the middle of the maze and had 60 s to find the target hole, from which the escape box had been removed. The cumulative distance from the target hole, total distance traveled, time to find the target hole, and velocity were calculated using Noldus Ethovision XT software and analyzed to assess spatial memory and learning. Cumulative distance is measured as the sum of the distance between the center point of the mouse and the center of the target hole over every sample from each trial at 30 samples per second. This distance stops accumulating when the trial ends either when time has elapsed or when the mouse enters the target box. Data are presented as the raw values of the cumulative distance.

### Anxiety Assessment

Anxiety-like behavior was assessed using the EPM 1 day before euthanasia. Mice were placed in the middle of the plus-shaped maze elevated 80 cm above the ground with two open and two closed arms perpendicular to each other in a brightly lit room. Animal movement was recorded during a 5 min trial. Each animal underwent only one trial. The center point of the mouse determined by Ethovision tracking was used to decide the current arm of the maze the mouse occupied. The time spent in open vs. closed arms was calculated.

### Euthanasia

Animals were euthanized on the next day after the last injection (22 days post-last injury/sham). Mice were anesthetized with 3% isoflurane and perfused transcardially with PBS, pH 7.4. After perfusion, the brains were post-fixed in a solution of 4% paraformaldehyde (PFA) at 4°C for 48 h and paraffin-embedded for immunohistochemistry.

### Tissue Processing

Brain samples fixed in PFA were processed in paraffin using the Tissue-Tek VIP (Sakura, Torrance, CA, USA). Sagittal sections were cut at 8 μm using a Leica RM2235 microtome and mounted on positively charged glass slides. Prior to staining, sections were deparaffinized in xylene and rehydrated in ethanol solutions of decreasing concentrations.

### Immunohistochemistry

#### Non-fluorescent Staining

##### GFAP

Following rehydration, slides were processed with hydrogen peroxide for 15 min and heated in the citric acid buffer (pH 6) for antigen retrieval. Slides were then blocked with normal goat serum, washed with PBS, and incubated in a primary antibody for GFAP overnight at 4°C (GFAP7857983, Aves Labs, Inc., 1:10,000). On the next day, slides were processed using the anti-chicken VectaSTAIN ABC Kit and developed with 3,3’-Diaminobenzidine (DAB) before mounting.

##### Iba1

Reactive microglia were stained using an anti-Iba1 antibody (ab107159, Abcam). After rehydration, slides were processed with hydrogen peroxide for 15 min followed by antigen retrieval using citric acid buffer (pH 6). Next, slides were blocked with rabbit serum for 1 h at room temperature and then incubated with the primary antibody (1:1,000) overnight. On the next day, samples were processed using the anti-goat VectaSTAIN ABC Kit and developed with DAB.

#### Fluorescent Staining

##### S100β/PSyk/Iba1/RZ3/CP13

Fluorescent staining was performed with the antibodies for microglial marker Iba1 (ab107159, Abcam), astroglial marker S100β (ab41458, Abcam), phosphorylated tau at Thr231 (RZ3) and at Ser202 (CP13), and PSyk (Tyr525/526; 2710S, Cell Signaling). Following rehydration, antigen retrieval was performed by heating slides in citric acid buffer for 7 min in a microwave oven. Next, slides were washed with PBS and transferred to a Sudan Black solution for 15 min to prevent autofluorescence. Slides were then blocked for 1 h with 10% donkey serum solution in PBS, and primary antibodies for Iba1 (1:300), S100β (1:500), RZ3 (1:400), CP13 (1:400) and PSyk (1:200) were applied overnight. On the next day, secondary antibodies AlexaFluor488 (A21202, Life Technologies), AlexaFluor568 (ab175477, Abcam), AlexaFluor 488 and AlexaFluor647 (A21449, Life Technologies) were applied for PSyk, Iba1, RZ3, CP13 and s100β respectively. Slides were mounted with ProLong Gold Antifade 4’,6-diamidino-2-phenylindole (DAPI) Mount. Each marker was stained separately except for the double staining for Iba1/PSyk that was performed to evaluate colocalization of the two.

### Imaging

Imaging of *non-fluorescent* samples stained for GFAP/Iba1 was performed on an Olympus DP72 microscope at 10× magnification. Further analysis of the images included quantification of GFAP and Iba1 signal using ImageJ. Images were separated into individual color channels (hematoxylin counterstain and DAB chromogen) using the color deconvolution algorithm (Ruifrok and Johnston, 2001). Three nonoverlapping regions of interest (ROI) of 100 μm^2^ per image were then selected for the hippocampus, the body of the corpus callosum (CC), and the cortex. A coverage area (%) per ROI was calculated, and the mean value for each animal was used for further statistical analysis.

*Fluorescent imaging* was perform using a confocal microscope (LSM 800 Zeiss) at 63× magnification. Cerebellum, hippocampus, and cortex were screened to find areas of interest such as activated microglia, activated astroglia, p-tau, and accumulation of PSyk. Z-stacks were recorded for every image and orthogonal projections were obtained to enable a 3D representation of the picture.

### MSD Multi-spot Assay

Cytokines profile was assessed using Proinflammatory Panel 1 (mouse) kit (V-PLEX K15048D) using cortex homogenates extracted from mice on day 22 post last mTBI. First, the calibration solution Diluent41 was prepared according to the kit protocol. Samples and controls were diluted 2-fold in Diluent41. Then, a combined detection antibody solution was prepared by mixing each antibody 5-fold with Diluent45. The plate was thoroughly washed three times with Wash Buffer and loaded with samples (50 μL/well) followed by a 2 h incubation at room temperature. Then, the plate was washed three times and detection antibody solution was added to samples at 25 μL/well. After a 2 h incubation, the plate was washed again with Wash Buffer and 2× Read Buffer T was added at a concentration 150 μL/well followed by plate analysis on the MSD instrument (MESO Quick Plex SQ120).

### Statistical Analysis

All experimental data were analyzed using JMP 12 Software. The data were checked for normality using Skewness-Kurtosis and Goodness of Fit. If normal, parametric methods (*t*-test and one-way ANOVA) were applied to calculate the significance between different experimental groups (*p-values* less than 0.05 were considered significant). The Shapiro-Wilk test was used if data were not normally distributed. All data were transformed to logarithm or square root, when required, to reach normality before further analysis. Repeated-measure analysis of variance (MANOVA) was used to analyze continuous performance of mice in the BM and Rotarod (*p* < 0.05 is significant). Error bars represent the standard error of the mean.

## Results

### Nilvadipine Mitigates r-mTBI-Induced Memory Deficits

**Figure 2:**
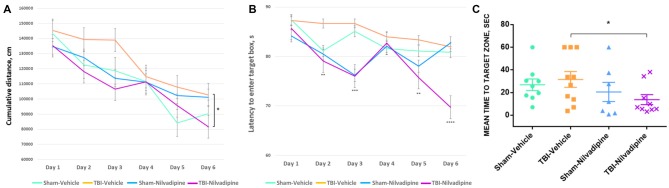
Evaluation of learning and spatial memory at 7 days post-last mild traumatic brain injury (mTBI) using BM. Acquisition data shows a learning trend in mice by the mean cumulative distance to the target hole in cm **(A)** and the mean time spent to enter the target hole in seconds **(B)**. All groups had similar learning rates. However, repetitive mTBI (r-mTBI)-Nilvadipine mice performed better than other groups (mean distance *p* < 0.05, multivariate analysis of variance (MANOVA); mean time: day 2 *p* < 0.01, day 3 *p* < 0.001, day 5 *p* < 0.01, day 6 *p* < 0.00001, one-way ANOVA). Probe data shows spatial memory by the mean time to locate the target hole (sec) after 6-day training. **(C)** r-mTBI-Nilvadipine group also performed better than r-mTBI-vehicle mice (*p* < 0.05, one-way ANOVA). *p < 0.5.

All animals showed a decrease of the cumulative distance to the target hole over the course of the acquisition trials. However, r-mTBI-vehicle mice performed worse compared to the sham controls (sham-vehicle) over a 6 day period (*p* < 0.05). Treatment with Nilvadipine decreased the cumulative distance of r-mTBI mice compared to the vehicle-treated r-mTBI mice (*p* < 0.001), indicating improved spatial learning (Figure 2A). The performance of sham-vehicle and sham-Nilvadipine mice were not different from each other (*p* > 0.5). Similar to the cumulative distance, the mean latency to enter the target box was decreased in Nilvadipine-treated vs. vehicle-treated r-mTBI mice (days 1 and 6 of acquisition: r-mTBI-Nilvadipine 85.7 s and 69.7 s r-mTBI-vehicle 87.3 s and 81.9 s *p* < 0.05; Figure 2B). Surprisingly, we didn’t see the difference in latency to enter the target box between groups on day 4. Behavioral testing data is inherently noisy and typically requires large *n* numbers to achieve statistical significance, thus the lack of an effect on latency during an individual day should not be interpreted as a lack of an overall effect during testing. Unexpectedly, probe data showed no difference in spatial memory between sham-vehicle, sham-Nilvadipine and r-mTBI-vehicle groups (*p* > 0.5; Figure 2C). However, r-mTBI-Nilvadipine mice showed significant improvements in finding the target hole compared to r-mTBI-vehicle animals (*p* < 0.05). Injured mice treated with Nilvadipine required less time overall (mean = 13.75 ± 6.9 s) to locate the target hole within the trial compared to other groups: r-mTBI-vehicle = 38.27 ± 4.4 s, sham-vehicle = 26.87 ± 5 s, sham-Nilvadipine = 20.51 ± 8.4 s. There was no significant effect of Nilvadipine treatment on motor function or anxiety level as measured by Rotarod (Supplementary Figure S1) and EPM (Supplementary Figure S2).

### Immunohistochemistry

**Figure 3:**
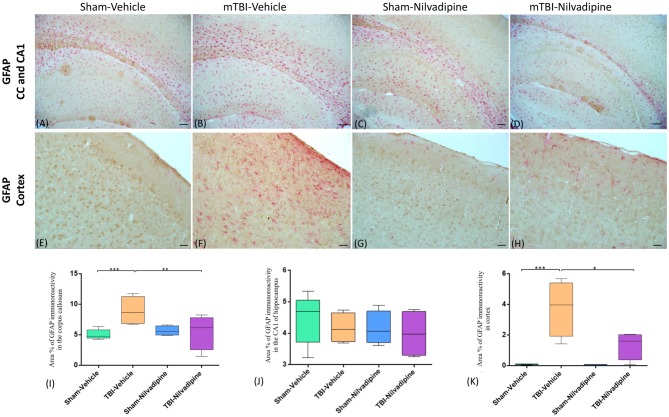
Evaluation of the effect of Nilvadipine after r-mTBI on astrocytes (GFAP) at 22 days post-r-mTBI in the corpus callosum (CC), CA1 area of hippocampus and cortex. **(A–D)** Representative images (10×) of the GFAP staining in the CC in different treatment groups show excessive gliosis after r-mTBI which was reduced by Nilvadipine. In the CA1 **(A–D)**, no difference between groups was observed. **(E–H)** r-mTBI caused an increase in GFAP immunoreactivity surrounding the injury site in cortex (*p* < 0.001) which was attenuated by Nilvadipine (*p* < 0.05). Quantitative analysis of GFAP staining in three 100 μm^2^ fields of the CC **(I)**, CA1 **(J)** and cortex **(K)** at 22 days post-injury confirms injury-induced glial activation in the CC (*p* < 0.001) and cortex (*p* < 0.001) and a Nilvadipine-dependent decrease of GFAP. Scale bars equal 100 μm for **(A–D)** and 50 μm for **(E–H)** images. Data are presented as mean ± standard error of the mean; significance is calculated using one-way ANOVA. *p < 0.5; **p < 0.01; ***p < 0.001.

**Figure 4:**
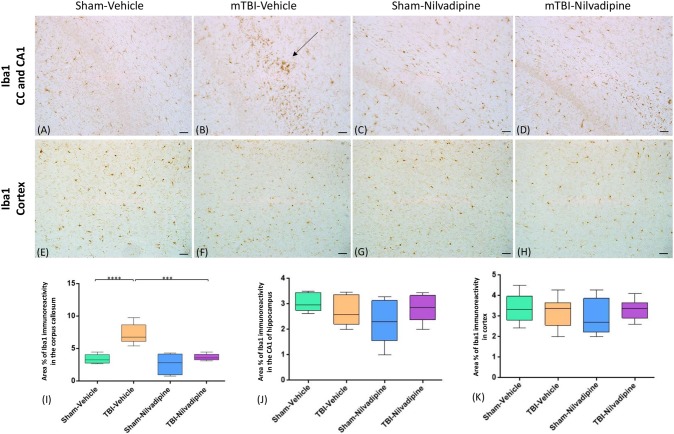
Evaluation of the effect of Nilvadipine after r-mTBI on microglial population (Iba1) at 22 days post-r-mTBI in CC, CA1 and cortex: **(A–D)** Representative images (20×) of the Iba1 staining in the CC revealed increased microglial activation after r-mTBI (*p* < 0.0001) and a reduction of Iba1 immunoreactivity by Nilvadipine (*p* < 0.001). No difference in Iba1 was detected for CA1 **(A–D)** and cortex **(E–H)**. Quantitative analysis of Iba1 staining in three 100 μm^2^ fields of the CC **(I)**, CA1 **(J)** and cortex **(K)** at 22 days post-injury confirms the injury-induced and Nilvadipine-induced changes in glial response in CC. Scale bars equal 50 μm. Data are presented as mean ± standard error of the mean; significance is calculated using one-way ANOVA. ***p < 0.001; ****p < 0.0001.

**Figure 5:**
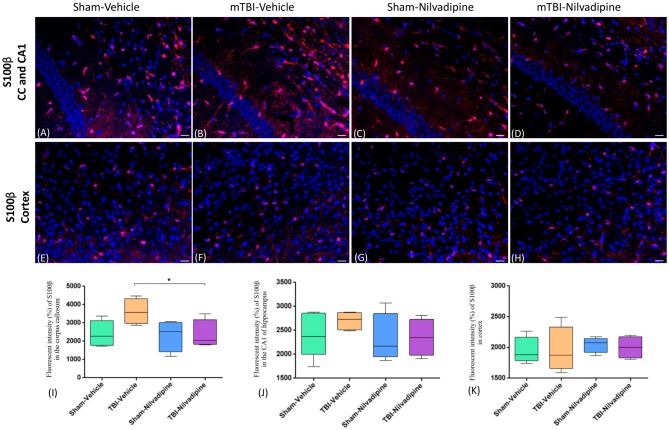
Fluorescent IHC analysis of S100β (red) in the CA1 are of hippocampus, CC and cortex. **(A–D)** Representative images of the S100β staining in CC in different treatment groups show an increased area of S100β immunoreactivity after r-mTBI (*p* > 0.05) which was reduced by Nilvadipine (*p* < 0.05). In the CA1, no difference between groups was observed (*p* > 0.05). **(E–H)** r-mTBI caused no effect on S100β intensity in cortex (*p* > 0.05). Scale bars equal 50 μm. Data are presented as mean ± standard error of the mean; significance is calculated using one-way ANOVA. **(I–K)** Quantitative analysis of the fluorescent intensity of S100β shows a Nilvadipine-dependant decrease of astrogliosis in the corpus callosum (*p* < 0.05) but not in the hippocampus and cortex. *p < 0.5.

**Figure 6:**
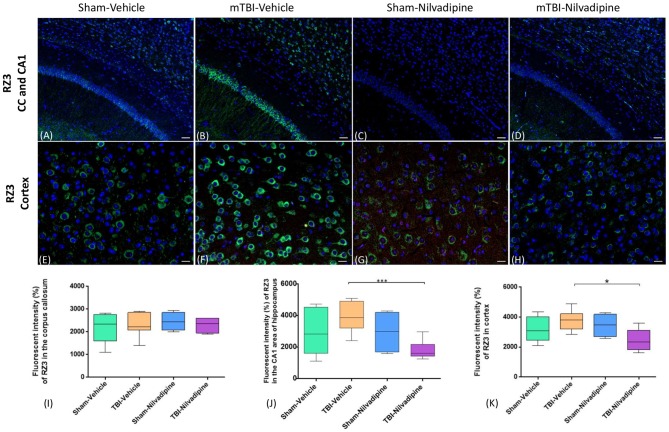
Fluorescent IHC analysis for pThr231 (RZ3; green)—in the CA1, CC and cortex. **(A–D)** In the CA1, IHC shows a trend of increased RZ3 immunoreactivity after r-mTBI which was decreased after treatment with Nilvadipine (*p* < 0.001) however no difference was observed in CC (*P* > 0.05). **(E–H)** In cortex, RZ3 intensity was slightly increased in r-mTBI-vehicle mice but not significant (*p* > 0.05). Similar to CA1, Nilvadipine reduced RZ3 signal in r-mTBI mice only. Quantitative analysis of the % intensity **(I–K)** shows that Nilvadipine reduces RZ3 fluorescent intensity in the CA1 by 50% (*p* < 0.001) and in cortex by 30% (*p* < 0.05). Scale bars equal 50 μm. Data are presented as mean ± standard error of the mean; significance is calculated using one-way ANOVA. *p < 0.5; ***p < 0.001.

**Figure 7:**
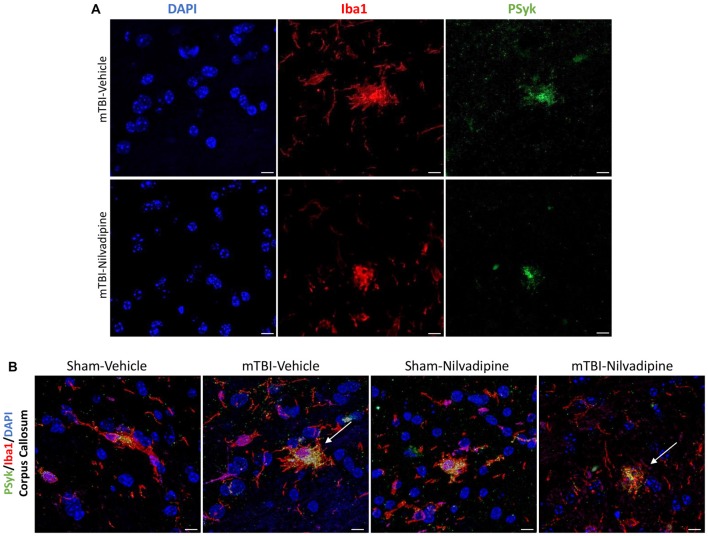
**(A)** Fluorescent immunohistochemistry for phosphorylated spleen tyrosine kinase (PSyk)/Iba1/4’,6-diamidino-2-phenylindole (DAPI) revealed a colocalization of PSyk and Iba1, indicating a strong link between activated Syk and neuroinflammation. **(B)** Immunofluorescent staining of the CC with DAPI, Iba1 and PSyk revealed an increased activity of PSyk in the r-mTBI-vehicle brains (white arrow). Upon treatment with Nilvadipine, PSyk signal was decreased to sham levels (white arrow). The mitigation of the PSyk signal was correlated with decreased Iba1. Scale bars equal 10 μm.

Consistent with our previous work, for mice subjected to r-mTBI, immunostaining for GFAP revealed evidence of a severe reactive astrogliosis in the CC (percent area: r-mTBI-vehicle 9.03% ± 1.05 vs. sham-vehicle 5.01% ± 1.2, *p* < 0.01; Figures 3A–D,I). However, no difference between groups was observed in the hippocampus (r-mTBI-vehicle 4.01% ± 0.5 vs. sham-vehicle 4.7% ± 0.65; *p* > 0.05; Figures 3A–D,J). After 21 days, significantly decreased GFAP immunoreactivity was observed in the CC (r-mTBI-Nilvadipine 6.3% ± 1.3 vs. r-mTBI-vehicle 9.03% ± 3.2; *p* < 0.01) of Nilvadipine r-mTBI vs. vehicle r-mTBI mice. Significant astrogliosis was also detected in the injured mice in the region of the cortex surrounding the injury site (percent area: r-mTBI-vehicle 4.02% ± 2.09 vs. sham-vehicle 0.08% ± 0.09, *p* < 0.001; Figures 3E–H,K). Treatment with Nilvadipine decreased GFAP immunoreactivity in r-mTBI mice (r-mTBI-Nilvadipine 1.75% ± 0.31 vs. r-mTBI-vehicle 4.02% ± 2.09; *p* < 0.05), but immunoreactivity was still significantly higher than the vehicle-sham level. A similar response was detected with anti-Iba1 staining, which indicated excessive TBI-dependent microgliosis in the CC (percent area: r-mTBI-vehicle 7.02% ± 2.2 vs. sham-vehicle 3.06% ± 0.7, *p* < 0.0001) and successful mitigation of this astrogliosis after treatment with Nilvadipine (percent area: r-mTBI-vehicle 7.02% ± 2.2 vs. r-mTBI-Nilvadipine 3.1% ± 2.5, *p* < 0.05; Figures 4A–D,I). No difference was observed for Iba1 in cortex (Figures 4E–H,K) and CA1 area of hippocampus (Figures 4A–D,J). However, in the CC, the r-mTBI-Nilvadipine group showed a notable decrease in the area of S100β immunoreactivity compared with their respective r-mTBI-vehicle controls (fluorescent intensity: r-mTBI-vehicle 3500% ± 500 vs. r-mTBI-Nilvadipine 2,354% ± 450, *p* < 0.05; Figures 5A–D,I). No differences were observed between groups in the CA1 area (Figure 5J) or cortex (Figures 5E–H,K). Fluorescent IHC for phospho-tau (RZ3) revealed no significant injury effect in the CA1 area of hippocampus (Figures 6A–D,J), CC (Figures 6A–D,I) and cortex (Figures 6E–H,K); nevertheless, there was a decreased immunoreactivity for RZ3 (p-Thr-231) in the CA1 region of hippocampus (*p* < 0.001; Figures 6A–D,J) and in cortex (*p* < 0.05; Figures 6E–H,K) after treatment with Nilvadipine compared to vehicle-treated mice. Quantitative analysis of the signal intensity confirms the reduction of RZ3 by 50% in the CA1 of r-mTBI-Nilvadipine (1,564% ± 107.6) vs. r-mTBI-vehicle mice (3,715% ± 430; Figure 6J). However, no difference between the groups was detected for CP13 in hippocampus, cortex, and CC (Supplementary Figure S3). Injured mice also demonstrated the presence of activated (phosphorylated) Syk compared to the sham-vehicle group. Qualitative analysis revealed accumulation of PSyk in certain sections of CC, but not uniformly across the brain. However, in these sections, PSyk was colocalized with reactive microglia (Iba1), indicating a strong link with neuroinflammation (Figure 7A). Treatment with Nilvadipine ameliorated PSyk and Iba1 in the r-mTBI mice (Figure 7B).

### Proinflammatory Cytokines Analysis

**Figure 8:**
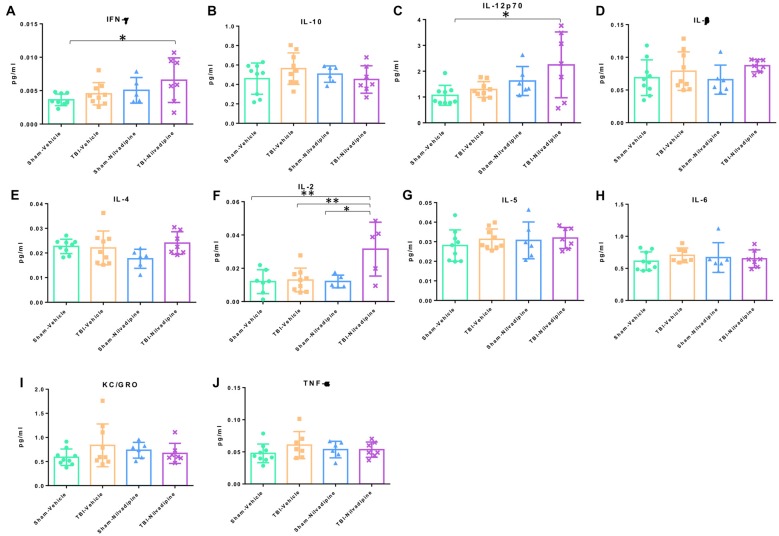
Quantitative analysis (pg/ml) of proinflammatory cytokines in cortex. The analysis showed a lack of r-mTBI cytokines behavior **(A–J)**. However, treatment with Nilvadipine caused an increase in IFN-γ **(A)**, IL-12p70 **(C)** and IL-2 **(F)** in the injured mice vs. sham-vehicle mice. Nilvadipine didn’t have an effect on cytokines in sham mice. Data are presented as mean ± standard error of the mean; significance is calculated using one-way ANOVA. *p <0.5; **p < 0.01.

In addition to IHC analysis of inflammatory markers, we performed an MSD analysis of the main proinflammatory cytokines: IFN-γ, IL-10, IL12p70, IL-β, IL-4, IL-2, IL-5, IL-6, KC/GRO and TBF-α. We observed that our model of r-mTBI in the aged hTau mice didn’t cause any effect compared to sham-vehicle mice at 21 days post injury (Figure 8). However, Nilvadipine increased the levels of IFN-γ, IL-12p70 and IL-2 compared to the sham-vehicle animals (Figures 8A,C,F).

## Discussion

The effect of TBI on aged animals and the ability of the aged brain to respond to the injury is remarkably understudied. Age is known to slow neurorehabilitation and prolong inflammation, which can worsen the pathogenesis of mTBI, exacerbate cognitive impairments, and increase the probability of developing dementia (Bartrés-Faz et al., 2001; Hebert et al., 2003; Primiani et al., 2014; von Bernhardi et al., 2015). Our previous studies showed that the anti-hypertensive dihydropyridine, Nilvadipine, is a Syk inhibitor which can decrease neuroinflammation and amyloid pathology in the PSAPP transgenic mice and tau phosphorylation in the P301S mouse model of tauopathy (Paris et al., 2014). Here, we tested the effects of Nilvadipine in hTau mice with r-mTBI to assess its effect on pathological and cognitive outcomes post-injury.

With regard to the neuropathological findings, consistent with our previous work using this r-mTBI paradigm, we observed increased GFAP and Iba1 immunoreactivity in the CC and in the area of the cortex beneath the impact site, even in these significantly aged mice. Our previous studies, wherein we have injured mice ranging from 3 months to 18 months old, have demonstrated robust r-mTBI-dependent neuroinflammation regardless of the age of the mice at the time of injury (Ojo et al., 2013; Mouzon et al., 2014; Mouzon B. et al., 2018; Lynch et al., 2016; Ferguson et al., 2017). Both young and aged groups of injured mice are characterized by reactive astrogliosis, and microgliosis in the CC, hippocampus, and cortex. Our data confirm the persistence of TBI consequences over the lifespan of mice injured young, and despite the inherent age-dependent increases in neuroinflammation an injury effect is evident even in very aged mice. However, no injury effect was observed with our panel of proinflammatory cytokines in cortex in our model of r-mTBI. We also did not observe any significant increase in tau phosphorylation following r-mTBI. Previous findings showing that tau phosphorylation increases with age in hTau sham animals (Andorfer et al., 2003; Polydoro et al., 2009; Ojo et al., 2013) are corroborated by our recent study which showed an overall increase of phosphorylated tau in aged (12 months old) when compared to our previously-studied young (3 months old) hTau mice (Ferguson et al., 2017). Thus, we hypothesize that the age-dependent increases in tau pathology may be obscuring any additional increases due to r-mTBI.

Regarding behavioral data, we did not detect an injury effect on motor function or anxiety-like behavior, and only a minor effect on spatial memory, which is likely due to the much older age of the animals at the time of injury in this study compared to our previous work. Our previous studies with the same injury paradigm in younger animals, aged between 3 months and 12 months old at the time of injury, resulted in memory impairment after r-mTBI (Mouzon et al., 2012, 2014; Ferguson et al., 2017). However, our data also show that in mice injured at a young age (2–3 months) cognitive deficits are less apparent over time, particularly when a year or more has elapsed post-injury (Mouzon et al., 2014; Ferguson et al., 2017; Mouzon B. C. et al., 2018). Thus, aging plays a confounding role in the baseline behavioral levels, so it obscures the effects of injury. Few studies have investigated the effects of r-mTBI on aged mice, and this study is the first to utilize this 5r-mTBI paradigm in such old mice. We propose that the advanced age of these animals causes detrimental deficits resulting in a “ceiling effect” that prevents a clear differentiation between age and injury-dependent deficits. In support of this hypothesis, studies of hTau vs. WT mice have shown that the hTau mice develop age-dependent memory deficits, not present at 4 months, but evident by 12 months of age (Polydoro et al., 2009; Yin et al., 2016). The characteristics of hTau mice aged 24 months have not previously been reported, but we would reasonably conclude that such mice would have dramatically impaired cognitive performance. Indeed, even in comparing sham mice across our studies, we observed decreased performance in cognitive tasks with age; e.g., in our study of hTau mice aged 3 or 12 months, cognitive performance was diminished in the older vs. young sham animals (Ferguson et al., 2017); while in cohorts of WT mice aged to 27 months, we saw a progressive worsening of memory throughout life (Mouzon B. C. et al., 2018). Thus, the age-dependent pathology underlying spatial memory (not specifically investigated here) appears sufficient to mask any additional effects of injury. Simen and colleagues reviewed a plethora of clinical and preclinical studies utilizing monkeys, rats and mice, and confirmed a decrease in episodic memory and other cognitive functions with age across species (Simen et al., 2011). These changes are believed to be associated with, but not limited to, age-dependent neuroinflammation (Gemma et al., 2005; Wan et al., 2007; Barrientos et al., 2009), demyelination (Vanguilder et al., 2012), impairment of synaptic receptors (Pagnussat et al., 2015), and increase of Ca^2+^ in the hippocampus (Foster, 2012). Such a diverse spectrum of age-related pathology will require much more work to understand any compounding effects of r-mTBI in the elderly. Nevertheless, our model demonstrates a clear TBI-dependent neuroinflammatory response, and although we do not observe increased tau phosphorylation or increased cognitive deficits following TBI, Nilvadipine treatment resulted in significant improvement in all three of these domains. Nilvadipine significantly decreased neuroinflammation in the CC and cortex, as shown by lower levels of astrogliosis and microgliosis. In addition, in r-mTBI mice, Nilvadipine significantly increased IFN-γ, IL-12p70 and IL-2, the only molecules from the analyzed spectrum of cytokines (Figure 8) exhibiting both pro- and anti-inflammatory properties. We believe these Nilvadipine-dependent increases in cytokine activation are important for modulating the inflammatory response after TBI. Schwulst et al. (2013) demonstrated that CCI leads to a significant decrease of IL-12 in blood up to 60 days post injury. They believe that it plays a crucial role in sustaining hypo-immune phenotype leading to chronic deteriorations. Another study showed that a suppression of IL-12p70, and not other members of IL-12 family, is linked to demyelination while an increase in IL-12p70 reversed the process (Lee et al., 2017). Interestingly, IL-12 family including IL-12p70 are important for the activation of IFN-γ supporting a strong link between the two in inflammation (Gee et al., 2009). IFN-γ has been shown to exhibit anti-inflammatory properties via several mechanisms: by inducing an expression of cytokine antagonists IL-1Ra and IL-18BP that are natural inhibitors of IL-1 and IL-18 accordingly (Sihvola and Hurme, 1989; Schindler et al., 1990; Arend et al., 1998; Dinarello et al., 1998; Dinarello, 2000; Mühl and Pfeilschifter, 2003); by inducing an expression of the SOCS (Suppressors of Cytokine Signaling) proteins (Alexander et al., 1999; Alexander, 2002); by inducing apoptosis (Spanaus et al., 1998; Refaeli et al., 2002) and by suppressing IL-8 expression (Gusella et al., 1993; Schnyder-Candrian et al., 1995). In addition, IL-2 was shown to inhibit differentiation of T cells into Th17 that promote inflammation and induce Treg expression which is important for cell survival and proliferation (Hoyer et al., 2008). In the TBI mice, administration of IL-2 complex was shown to suppress inflammation (by decreasing pro- and elevating anti-inflammatory cytokines), reduce tissue loss and increase neurological recovery (Gao et al., 2017).

Moreover, PSyk accumulation was colocalized with activated microglia in the CC in the r-mTBI-vehicle mice, and Nilvadipine treatment reduced both PSyk and Iba1 signals, to the sham levels. The neurobehavioral deficits in the mice correlate with tau phosphorylation, but Nilvadipine did not have a significant effect on cognitive performance in the sham mice, which exhibited similarly high levels of tau phosphorylation. Thus, it seems that the inflamed post-r-mTBI environment was necessary for the Nilvadipine to exhibit therapeutic properties. As mentioned previously, the Syk regulates neuroinflammation through the NFkB activity and also phosphorylates tau directly at Tyr18 (Paris et al., 2014). Nilvadipine inhibits Syk, and this also activates PKA which phosphorylates Ser9 of GSK3β, thereby inactivating this potent tau kinase. The Syk inhibitory properties of the Nilvadipine have been previously demonstrated in mouse models of AD and tauopathies (Paris et al., 2014), but more work is required to confirm that this is the mechanism through which it elicits the favorable responses described in this study. Nilvadipine is also an L-type Ca^2+^ channel blocker (Peters et al., 2015) and similar compounds have been investigated in TBI clinical trials (albeit these were more severe TBI cases) with very confounding results (Xu et al., 2013). We have previously shown efficacy of the non-blood-pressure-lowering enantiomer of Nilvadipine ((-)-Nilvadipine) in the CCI mouse model of TBI (Ferguson, pers. comm). Determining whether or not this enantiomer has potency in this mTBI model is among the studies we propose in order to elucidate the mechanism of action and pursue Nilvadipine’s potential as a therapeutic for r-mTBI. In addition, our next step will be to repeat the study with younger hTau mice to determine the role of aging in r-mTBI pathology and treatment. It will not only help to explain current results but also to demonstrate an importance of age for developing therapeutic strategies for r-mTBI.

## Author Contributions

AM performed experiments on mice, analyzed data and prepared the initial draft of the manuscript. SF set up the behavior room and performed behavior data interpretation. BM supervised immunohistochemistry analysis. CL performed injuries. NS and BM performed immunohistochemistry. MM revised the manuscript. FC conceived and designed the research. FC is a VA Research Career Scientist and FC, BM and SF are CENC investigators. DP has provided Nilvadipine, helped with designing a treatment protocol and the analysis of PSyk, and participated in revising the manuscript. All authors reviewed the final manuscript and approved its publication.

## Conflict of Interest Statement

The authors declare that the research was conducted in the absence of any commercial or financial relationships that could be construed as a potential conflict of interest.
