# Supplementary figures and images for: Treatment With Nilvadipine Mitigates Inflammatory Pathology and Improves Spatial Memory in Aged hTau Mice After Repetitive Mild TBI
Source: Front Aging Neurosci. 2018 Oct 11;10:292. doi: 10.3389/fnagi.2018.00292 (PMC6193195; doi:10.3389/fnagi.2018.00292)

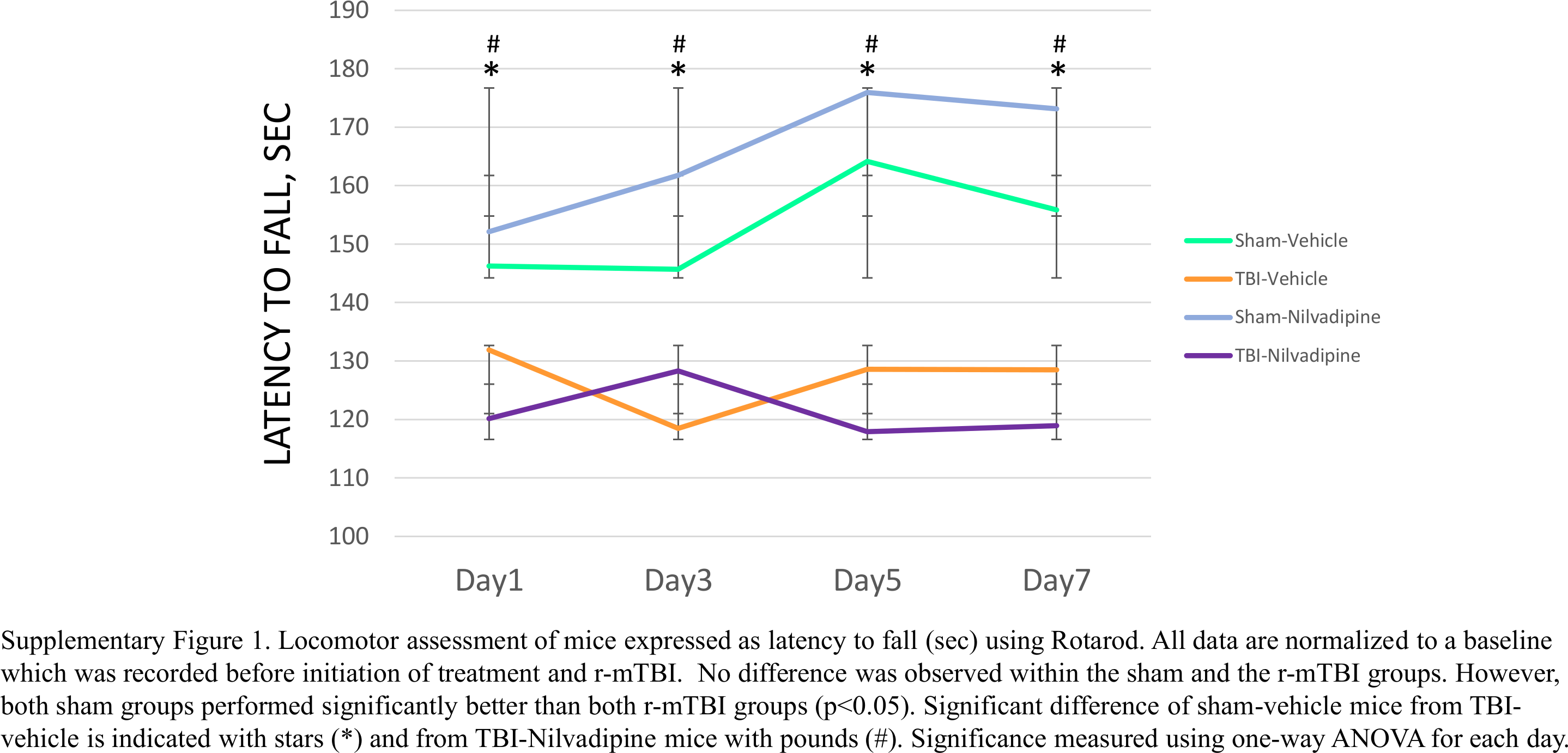

Supplement: Supplementary file 1 [file Image_1.TIFF]

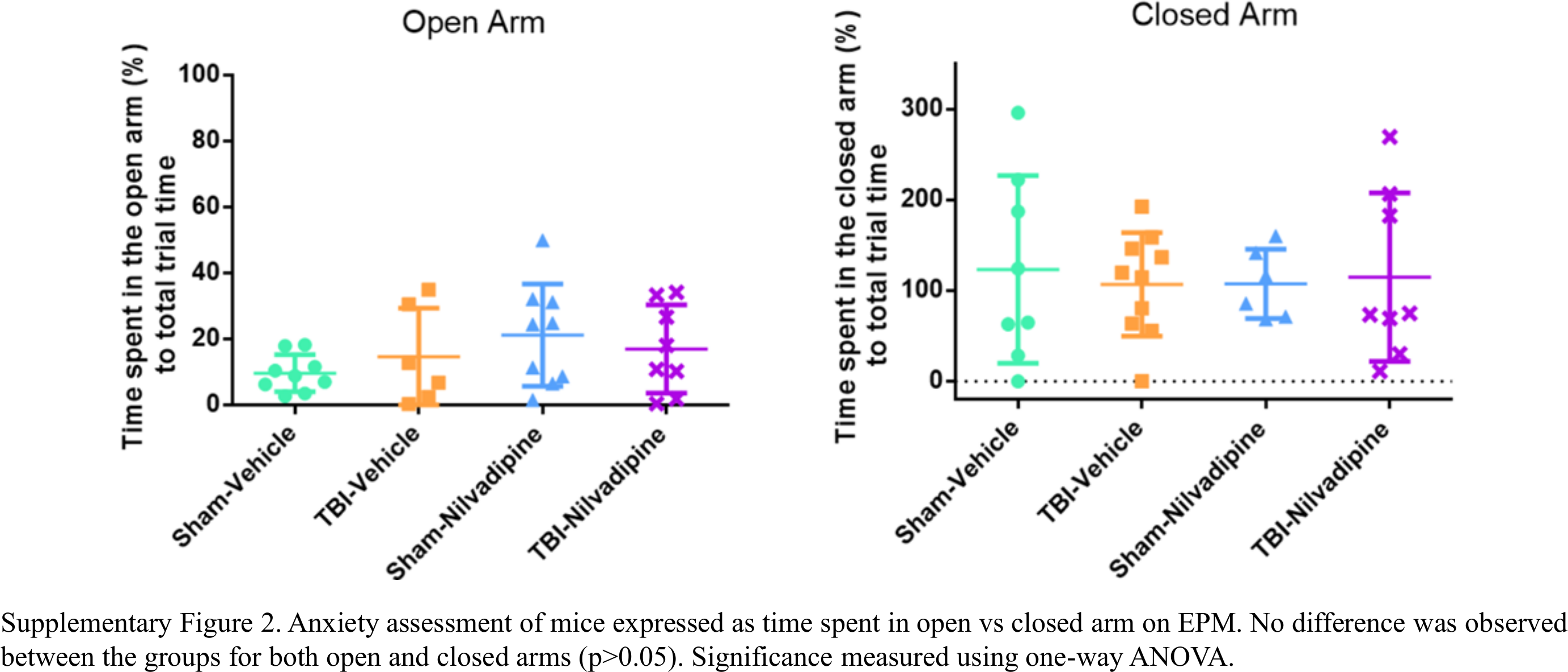

Supplement: Supplementary file 2 [file Image_2.TIFF]

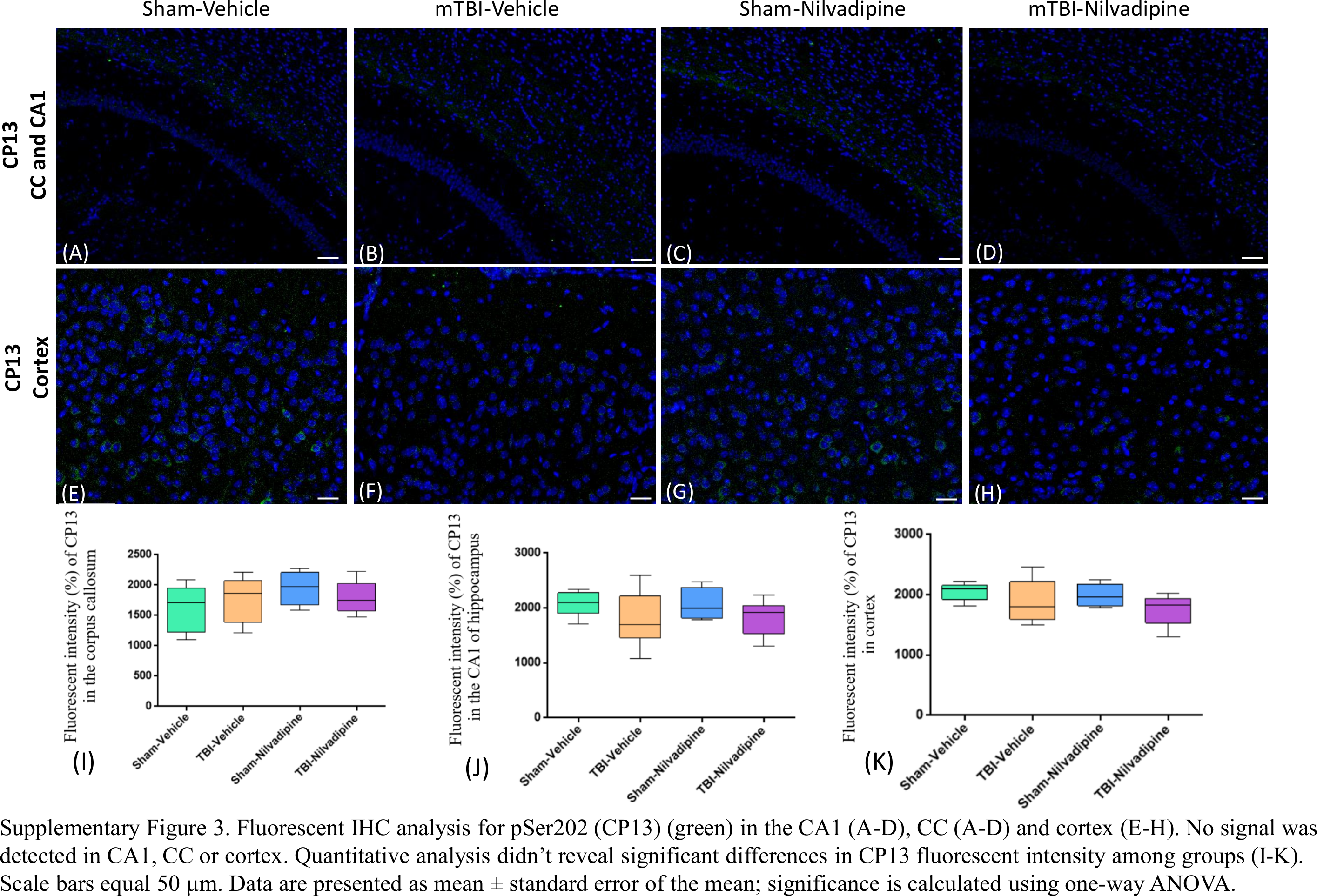

Supplement: Supplementary file 3 [file Image_3.TIFF]
